# Comparison of Sevoflurane and Desflurane on Hepatocellular Carcinoma Recurrence After Living Donor Liver Transplantation: A Propensity Score-Matched Analysis

**DOI:** 10.3390/medicina62050876

**Published:** 2026-05-03

**Authors:** Hyeun-Joon Bae, Sa-Jin Kang, Kyoung-Sun Kim, Hye-Mee Kwon, In-Gu Jun, Jun-Gol Song, Gyu-Sam Hwang

**Affiliations:** Department of Anesthesiology and Pain Medicine, Asan Medical Center, University of Ulsan College of Medicine, 88 Olympic-ro 43-gil, Songpa-gu, Seoul 05505, Republic of Korea; joshpheonix1@gmail.com (H.-J.B.);

**Keywords:** living donor liver transplantation, hepatocellular carcinoma, volatile anesthetics, tumor recurrence

## Abstract

*Background and Objectives*: While liver transplantation (LT) is a definitive treatment for hepatocellular carcinoma (HCC), tumor recurrence remains a major clinical concern. Although volatile anesthetics influence oncological outcomes, direct comparison between sevoflurane and desflurane remains scarce. This study aimed to investigate the impact of the recipient’s volatile anesthetic choice (sevoflurane vs. desflurane) on HCC recurrence following living donor liver transplantation (LDLT). *Materials and Methods*: This retrospective cohort study included adult patients who underwent LDLT for HCC. Patients were then divided into sevoflurane and desflurane groups, and propensity score matching (PSM) was used to balance baseline variables. The primary outcome was HCC recurrence, and the secondary outcomes were overall survival (OS) and postoperative C-reactive protein (CRP) levels as a marker for the postoperative systemic inflammatory response. *Results*: After PSM, 373 matched pairs (*n* = 746) were analyzed. HCC recurrence was significantly higher in the sevoflurane group compared to the desflurane group (19.6% vs. 13.1%, *p* = 0.023). Kaplan–Meier analysis also demonstrated that cumulative recurrence of HCC was significantly higher in recipients who received sevoflurane anesthesia than in those who received desflurane (log-rank *p* = 0.0018). In multivariate Cox proportional hazards regression analysis, the use of sevoflurane for anesthesia maintenance was an independent risk factor forHCC recurrence (Hazard Ratio, 1.66; 95% Confidence Interval, 1.15–2.39; *p* = 0.007). Regarding OS, no significant difference was observed between the two groups (log-rank *p* = 0.1123). Postoperative CRP levels were significantly higher in the sevoflurane group compared to the desflurane group, suggesting a more intense systemic inflammatory response associated with sevoflurane maintenance. *Conclusions*: For HCC patients undergoing LDLT, anesthesia maintenance with desflurane is associated with a reduced risk of tumor recurrence compared to sevoflurane, without a significant impact on overall survival.

## 1. Introduction

Hepatocellular carcinoma (HCC) is a leading indication for liver transplantation (LT), accounting for approximately 20–40% of all procedures performed worldwide [1,2]. Despite the application of selection criteria such as the Milan or University of California San Francisco (UCSF) criteria, HCC recurrence occurs in 8% to 20% of recipients [3,4]. Various clinicopathological factors—including tumor burden (size and number of nodules), microvascular invasion, and histological grade—along with serum biomarkers such as alpha-fetoprotein (AFP) and protein induced by vitamin K absence or antagonist-II (PIVKA-II), significantly influence the risk of recurrence [5,6]. In addition to these predictors, identifying modifiable clinical factors is crucial to develop strategies that improve post-transplant survival independently of tumor-related characteristics.

Recent studies suggest that the choice of anesthetic technique can influence the risk of tumor recurrence after surgery [7,8]. Surgical trauma induces a profound stress response that suppresses cell-mediated immunity, thereby creating a favorable microenvironment for the survival and metastasis of circulating tumor cells [9,10]. Thus, the choice of anesthetic agents may mitigate this surgical stress and immune suppression. In addition, experimental studies have shown the potential tumor-suppressive properties of volatile anesthetics, with evidence suggesting that these agents can exert direct anti-tumor effects by inhibiting cancer cell proliferation, migration, and invasion through various molecular pathways [11,12].

Most clinical studies have primarily focused on broad comparisons between propofol-based total intravenous anesthesia (TIVA) and volatile anesthetics [13,14,15]. Given their distinct effects on oncogenesis, the individual impacts of sevoflurane and desflurane should be evaluated independently [16,17]. Furthermore, in the context of LT, which involves significant surgical stress and inevitable immunosuppression, clinical evidence evaluating the oncological impacts of volatile anesthetics in recipients remains limited. Therefore, this study aimed to investigate the impact of volatile anesthetic choice (sevoflurane versus desflurane) on HCC recurrence and overall survival (OS) in living donor liver transplantation (LDLT) recipients.

## 2. Materials and Methods

### 2.1. Patients

This retrospective cohort study investigated adult patients (age ≥ 18 years) who underwent LDLT for HCC at a major transplant center in South Korea between January 2008 and December 2025. Patients were excluded based on the following criteria: (1) combined use of sevoflurane and desflurane during anesthesia; and (2) administration of sevoflurane anesthesia to the living donor. Given that LDLT uniquely involves both a donor and a recipient, variations in the donor’s anesthetic protocol can introduce unmeasured heterogeneity in baseline graft conditions, thereby acting as a potential confounding factor. To strictly evaluate the oncological effect of the recipient’s anesthetic choice, the analysis was restricted to a subcohort where all donors received desflurane [18]. Patients were then divided into recipient-sevoflurane and recipient-desflurane groups.

### 2.2. Anesthetic Management and Surgical Procedures

The anesthetic management and surgical procedures for LDLT followed standard institutional protocols [19,20]. Anesthesia was induced with propofol, fentanyl, and rocuronium. Maintenance was achieved using either sevoflurane or desflurane, combined with continuous infusion of fentanyl and rocuronium. The choice between sevoflurane and desflurane was at the discretion of the attending anesthesiologist. Volatile anesthetic concentrations were titrated to maintain a Bispectral Index or SedLine value below 50. Standard hemodynamic monitoring included invasive arterial pressure monitoring via radial and femoral artery cannulation, and pulmonary artery catheterization for advanced assessment. Vasopressors, including norepinephrine, were administered to maintain a mean arterial pressure ≥ 65 mmHg.

The surgical procedure followed standard LDLT techniques. The native liver was mobilized and dissected with exposure of the inferior vena cava. The donor graft was implanted via hepatic vein anastomosis, followed by end-to-end portal vein anastomosis. Graft reperfusion was initiated after portal vein anastomosis completion. During reperfusion, hemodynamic changes were monitored, and bolus doses of vasopressors, calcium gluconate, or sodium bicarbonate were administered to stabilize hemodynamics and correct electrolyte or acid-base imbalances. Subsequently, sequential hepatic artery and bile duct anastomoses were performed. The detailed surgical techniques for LDLT have been previously described [21].

### 2.3. Clinical Data and Outcomes

Clinical data of donors and recipients were extracted from the electronic medical record system of our institution. Recipient demographic and clinical variables included age, sex, body mass index, diabetes mellitus, hypertension, chronic kidney disease, etiology of liver cirrhosis (hepatitis B virus, hepatitis C virus, and alcoholism), and the Model for End-Stage Liver Disease (MELD) score. Preoperative AFP and PIVKA-II were collected and categorized using established cutoffs (>400 ng/mL and >40 mAU/mL) based on previous literature [22,23]. C-reactive protein (CRP) levels were collected at baseline and daily from postoperative day (POD) 1 to 7. Donor and intraoperative variables consisted of donor age, donor sex, total fatty change, graft-to-recipient weight ratio, anesthesia time, and the incidence of massive transfusion (defined as ≥10 units of packed red blood cells within 24 h or ≥4 units within 1 h) [24]. Pathological and tumor characteristics included the Barcelona Clinic Liver Cancer (BCLC) stage, Milan and UCSF criteria, number of nodules, maximal tumor size, percentage of tumor necrosis, vascular or lymph node (LN) invasion, and positron emission tomography (PET) findings [25,26,27].

The primary outcome of this study was HCC recurrence. The secondary outcomes included OS and the perioperative systemic inflammatory response, assessed by CRP levels.

### 2.4. Statistical Analysis

Continuous variables were expressed as means ± standard deviations and compared using the Student’s *t*-test or Mann-Whitney U test, as appropriate. Categorical variables were presented as numbers and percentages and compared using the Chi-square test or Fisher’s exact test.

To minimize the impact of potential confounding factors and selection bias between the sevoflurane and desflurane groups, propensity score matching (PSM) was performed. Propensity scores were calculated using a logistic regression model incorporating comprehensive baseline covariates. Patients were matched in a 1:1 ratio using the nearest-neighbor method with a caliper width of 0.1. The balance of covariates was assessed using the standardized mean difference, with a value less than 0.1 indicating a well-balanced distribution.

The incidences of tumor recurrence and all-cause mortality were expressed as the number of events and events per 100 person-years. Cumulative recurrence and cumulative mortality rates were compared between the groups using Kaplan–Meier analysis and the log-rank test. To identify independent predictors of tumor recurrence and OS, univariate and multivariate Cox proportional hazards regression analyses were conducted. Variables with a *p*-value < 0.1 in the univariate analysis were incorporated into the multivariate model, and a backward stepwise elimination procedure based on the Akaike Information Criterion was used to determine the final predictive model. To adjust for potential time-era bias over the 18-year study period, transplantation year was included as a covariate in the multivariate Cox models. For postoperative CRP trend analyses, longitudinal changes between groups from POD 1 to 7 were evaluated using a linear quantile mixed model (LQMM). An interaction term between time and group (P for interaction) was included to assess differences in the trajectory of CRP levels. Daily comparisons from POD 1 to 7 were conducted via post-hoc analysis of the LQMM, while point-wise comparisons at Baseline and for the recorded maximum CRP values were performed using the Mann-Whitney U test. Additionally, the predictive performance of the maximum postoperative CRP level for HCC recurrence was evaluated using the concordance index (C-index). All statistical analyses were performed using R software (version 4.5.1; R Foundation for Statistical Computing, Vienna, Austria).

### 2.5. Ethical Statements

Approval of this study was obtained from the Institutional Review Board of our center (protocol number: 2026-0334 and date of 13 March 2026). Given the retrospective design, the board waived the necessity for obtaining informed consent from the participants.

## 3. Results

### 3.1. Study Population and Propensity Score Matching Analysis

**Figure 1:**
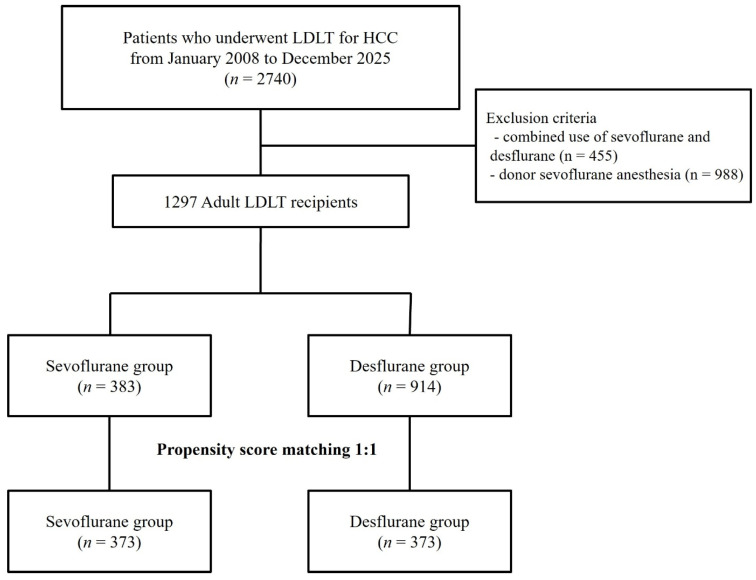
Flowchart of the study population. Abbreviations: LDLT, living donor liver transplantation; HCC, hepatocellular carcinoma.

A total of 2740 patients who underwent LDLT for HCC between January 2008 and December 2025 were initially screened (Figure 1). After applying the exclusion criteria, a total of 1297 recipients were included in the initial unmatched cohort. Among these, 383 patients received sevoflurane and 914 received desflurane for anesthesia maintenance. The baseline characteristics of the unmatched cohort are summarized in Table 1. Prior to PSM, the two groups exhibited significant differences in several baseline variables. PSM (1:1 ratio) resulted in 373 matched pairs, achieving well-balanced baseline characteristics between groups.

### 3.2. Primary Outcome: Hepatocellular Carcinoma Recurrence

**Figure 2:**
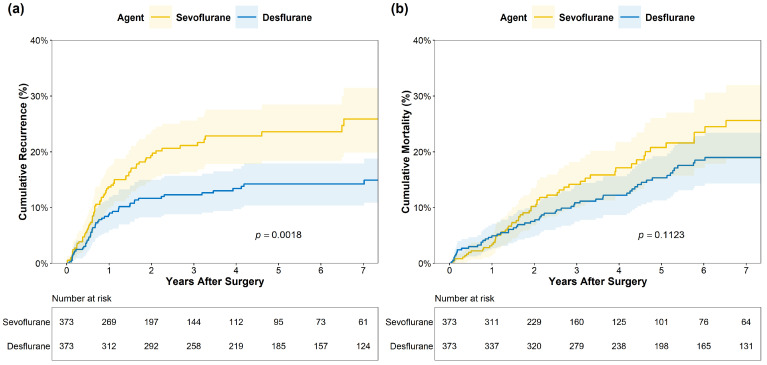
Kaplan–Meier curves for (**a**) cumulative recurrence of hepatocellular carcinoma (HCC) and (**b**) cumulative mortality according to the recipient’s volatile anesthetic choice. The cumulative incidence of HCC recurrence and all-cause mortality are compared between the sevoflurane group (yellow line) and the desflurane group (blue line) in the propensity score-matched cohort. The shaded areas represent the 95% confidence intervals. The *p*-values were calculated using the log-rank test. The number of patients at risk at each time point is provided in the tables below the graphs.

In the propensity score-matched cohort, HCC recurrence was observed in 73 patients (19.6%) in the sevoflurane group and 49 patients (13.1%) in the desflurane group (*p* = 0.023) (Table 2). The incidence rate of recurrence was significantly higher in the sevoflurane group than in the desflurane group (13.7 vs. 8.7 per 100 person-years, *p* = 0.039). The 1-year cumulative recurrence rates were also significantly higher in the sevoflurane group (5.3%) compared to the desflurane group (2.7%, *p* < 0.001). Kaplan–Meier analysis also demonstrated that cumulative recurrence of HCC was significantly higher in recipients who received sevoflurane anesthesia than in those who received desflurane (log-rank *p* = 0.0018) (Figure 2a). Regarding the anatomical distribution of the first documented recurrence, the most frequent sites were the lungs and the liver graft, followed by lymph nodes and bones.

Univariate and multivariate Cox proportional hazards regression analyses were performed to identify independent risk factors for HCC recurrence (Table 3). In the univariate analysis, factors significantly associated with recurrence included the use of sevoflurane, beyond Milan criteria, AFP > 400 ng/mL, PIVKA-II > 40 mAU/mL, hypermetabolism findings on PET, maximal tumor size, tumor necrosis > 30%, vascular or LN invasion, and transplant year. In the multivariate analysis, the choice of sevoflurane as the recipient’s anesthetic agent remained a significant independent predictor of HCC recurrence, associated with a 66% increased risk compared to desflurane (Hazard Ratio [HR], 1.66; 95% Confidence Interval [CI], 1.15–2.39; *p* = 0.007). Furthermore, the multivariate analysis identified PIVKA-II > 40 mAU/mL (HR, 1.61; 95% CI, 1.08–2.40; *p* = 0.018), hypermetabolism findings on PET (HR, 1.88; 95% CI, 1.26–2.79; *p* = 0.002), maximal tumor size (HR, 1.11; 95% CI, 1.02–1.21; *p* = 0.017), vascular or LN invasion (HR, 3.08; 95% CI, 2.03–4.67; *p* < 0.001), and transplant year (HR, 0.93; 95% CI, 0.89–0.98; *p* = 0.004) as significant independent predictors of HCC recurrence.

### 3.3. Secondary Outcome

Regarding OS, no significant differences were observed between the sevoflurane and desflurane groups in terms of total number of all-cause mortality (15.5% each, *p* = 1.000), incidence rate (3.87 vs. 2.93 per 100 person-years, *p* = 0.134), and 1-year cumulative mortality (3.4% vs. 4.9%, *p* = 0.307). Kaplan-Meier analysis of the cumulative mortality rates showed no statistically significant difference between the two groups (log-rank *p* = 0.1123) (Figure 2b). In the multivariate Cox proportional hazards regression analysis, the use of sevoflurane for anesthesia maintenance was not an independent predictor of overall survival (Table 4; HR, 1.32; 95% CI, 0.91–1.91; *p* = 0.145).

**Figure 3:**
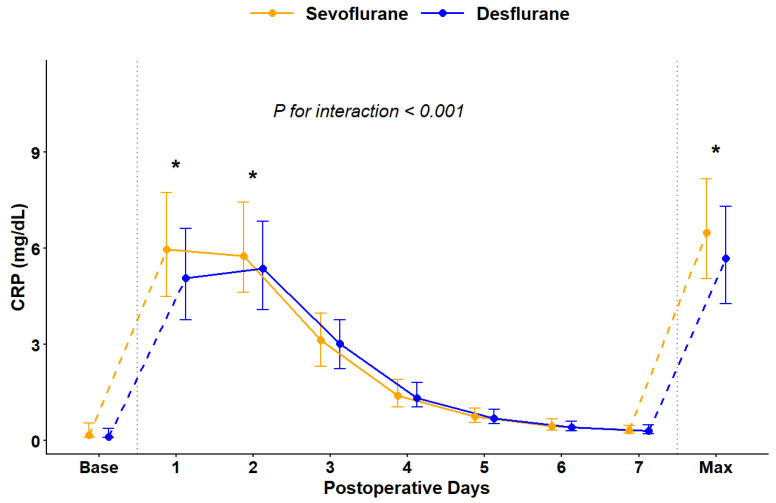
Baseline and postoperative trends of C-reactive protein (mg/dL) levels are compared between the sevoflurane group (yellow line) and the desflurane group (blue line). Data are presented as median with interquartile range (IQR). The ‘Max’ value represents the maximum CRP level recorded for each patient between postoperative day (POD) 1 and POD 7. The overall trend difference over time between the two groups was evaluated using a linear quantile mixed model (LQMM), with the P for interaction indicated on the graph. Asterisks (*) indicate a statistically significant difference (*p* < 0.05) between the two groups. Dotted vertical lines distinguish the baseline, postoperative daily trends, and the recorded maximum values. Abbreviations: CRP, C-reactive protein.

To evaluate the impact of volatile anesthetics on the systemic inflammatory response, perioperative trends in CRP levels were analyzed in the propensity score-matched cohort (Figure 3). While baseline CRP levels did not differ significantly between the groups, postoperative levels followed significantly different trajectories, with a significant time-by-group interaction (P for interaction < 0.001). Patients in the sevoflurane group exhibited significantly higher median CRP levels on POD 1 and 2 compared to those in the desflurane group. Additionally, the maximum CRP level during the first postoperative week was significantly greater in the sevoflurane group than in the desflurane group. Furthermore, to determine the prognostic relevance of this inflammatory response, we assessed the predictive performance of the maximum postoperative CRP level for HCC recurrence. The C-index for the association between postoperative CRP elevation and recurrence risk was 0.561 (95% CI, 0.510–0.612).

## 4. Discussion

In this retrospective cohort study, we investigated the impact of the recipient’s volatile anesthetic choice on HCC recurrence and OS following LDLT. Our results suggest that the use of sevoflurane for anesthesia maintenance in recipients was associated with a higher risk of HCC recurrence compared to desflurane, although no significant difference was observed in OS. To our knowledge, this is the first study to evaluate the impact of recipient volatile anesthetic choice on HCC recurrence in the context of LDLT.

Although surgical resection is the primary curative treatment for solid tumors, the resulting physiological stress can paradoxically promote tumor recurrence [28,29]. The surgical stress inherent in major surgeries, characterized by extensive tissue injury and prolonged manipulation, triggers a systemic neuroendocrine response via the hypothalamic-pituitary-adrenal axis and the sympathetic nervous system [30,31]. This activation results in elevated circulating levels of catecholamines and prostaglandins, both of which are known to impair host anti-tumor immune surveillance [32,33]. These mediators attenuate anti-tumor immunity by suppressing the cytotoxic activity of natural killer (NK) cells and limiting the proliferation of cytotoxic T lymphocytes [10,34]. In the setting of LT, this vulnerability is further compounded by the mandatory administration of immunosuppressive therapy [35].

Experimental studies show that volatile anesthetics possess potential anti-oncogenic and immune-preserving properties [11]. Specifically, sevoflurane inhibits tumor progression by inducing apoptosis and modulating the hypoxia-inducible factor-1alpha signaling pathway, while desflurane suppresses tumor cell invasion through the downregulation of matrix metalloproteinase-9 [12,36]. Furthermore, Woo et al. reported that desflurane maintains a favorable immunomodulatory profile, showing preserved NK cell counts and a stable T helper 1/T helper 2 ratio compared to propofol during the perioperative period [37]. However, since both sevoflurane and desflurane also exhibit pro-oncogenic properties, the interpretation of their net oncological impact is complex and context-dependent [38,39].

While substantial research has compared the oncological outcomes of propofol-based TIVA and volatile anesthetics, direct head-to-head comparisons between sevoflurane and desflurane remain remarkably scarce. Elias et al. found that sevoflurane was associated with a higher risk of recurrence than desflurane in patients with stage III epithelial ovarian cancer [40]. In contrast, a study by Yoshida et al. involving older patients undergoing major gastrointestinal surgery observed no significant differences in 1-year mortality or the deterioration of long-term care-need levels between desflurane and sevoflurane [41]. Given that sevoflurane and desflurane show distinct molecular influences on oncogenesis and perioperative immunity, evaluation of their impact independently across various malignancies is needed.

In this study, the sevoflurane group exhibited significantly higher postoperative CRP levels compared to the desflurane group. Elevation of postoperative CRP reflects the magnitude of surgical stress and tissue injury [42]. Previous studies have demonstrated that elevated CRP levels, both preoperative and postoperative, are independent predictors of poor overall survival and higher tumor recurrence rates in HCC [43,44,45]. Persistent systemic inflammation, often accompanied by elevated pro-inflammatory cytokines, is known to create a pro-tumorigenic microenvironment that facilitates the recurrence of residual cancer cells [46,47]. The relatively lower postoperative CRP levels observed in the desflurane group suggest that desflurane may exert a more favorable effect on modulating the inflammatory response during the perioperative period of LDLT. However, the modest predictive performance of maximum CRP (C-index, 0.561) suggests that systemic inflammation represents only one component of a multifactorial process. Further prospective studies are therefore warranted to fully investigate the complex mechanisms underlying HCC recurrence.

In this study, analyzing this large cohort enabled a comprehensive assessment of long-term oncological outcomes in patients undergoing LDLT for HCC. Unlike predetermined variables such as tumor biology or surgical complexity, the selection of a volatile anesthetic is a readily modifiable factor controlled by the anesthesiologist. Our findings suggest that selecting desflurane over sevoflurane for anesthesia maintenance might be considered a potential intraoperative strategy to reduce the recurrence of HCC after LDLT. However, this benefit did not translate into a significant improvement in overall survival. This discrepancy may be attributed to the censoring of data before a sufficient number of recurrence-related deaths occurred, or to the presence of competing risks of mortality unrelated to tumor recurrence.

Despite these clinical implications, our study has several limitations. First, the retrospective design introduces the potential for selection bias and unmeasured confounding factors. To mitigate these limitations and strengthen the validity of our findings, we employed a PSM analysis. Second, although a large cohort was used to ensure statistical power, the population was recruited from a single center, which may limit the generalizability of our findings, necessitating multi-center validation. Third, we did not investigate the dose-dependent effects of volatile anesthetics, although the anesthetic dose was titrated to maintain a target anesthetic depth monitored by electroencephalography-based indices. Analyzing such cumulative exposure could provide deeper insights into their potential oncological impact.

## 5. Conclusions

In conclusion, our findings show that selecting desflurane over sevoflurane for anesthesia maintenance in HCC patients undergoing LDLT may reduce the risk of tumor recurrence, although no significant impact was observed on overall survival. This protective effect against recurrence may be attributed to the more favorable modulation of the postoperative systemic inflammatory response, as evidenced by lower CRP levels in the desflurane group.

## Figures and Tables

**Table 1:** Baseline characteristics of the study population.

	Unmatched (*n* = 1297)				Matched (*n* = 746)			
	Sevoflurane (*n* = 383)	Desflurane (*n* = 914)	*p*	SMD	Sevoflurane (*n* = 373)	Desflurane (*n* = 373)	*p*	SMD
Demographic variables								
Age (years)	57.8 ± 8.1	57.8 ± 7.4	0.968	0.002	57.7 ± 8.2	57.7 ± 7.6	0.893	0.010
Sex, male	311 (81.2)	746 (81.6)	0.921	0.011	301 (80.7)	300 (80.4)	1.000	0.007
Body mass index (kg/m^2^)	24.5 ± 3.6	24.7 ± 3.5	0.425	0.048	24.6 ± 3.6	24.6 ± 3.4	0.882	0.011
Diabetes mellitus	130 (33.9)	263 (28.8)	0.075	0.112	124 (33.2)	121 (32.4)	0.876	0.017
Hypertension	110 (28.7)	256 (28.0)	0.848	0.016	105 (28.2)	105 (28.2)	1.000	<0.001
Chronic kidney disease	6 (1.6)	21 (2.3)	0.530	0.053	6 (1.6)	10 (2.7)	0.448	0.074
Etiology & lab results								
Hepatitis B virus	260 (67.9)	651 (71.2)	0.257	0.073	258 (69.2)	257 (68.9)	1.000	0.006
Hepatitis C virus	26 (6.8)	67 (7.3)	0.820	0.021	24 (6.4)	24 (6.4)	1.000	<0.001
Alcoholism	72 (18.8)	158 (17.3)	0.568	0.039	65 (17.4)	72 (19.3)	0.570	0.048
MELD score	12.0 ± 6.4	11.1 ± 5.3	0.017	0.140	11.7 ± 6.1	11.4 ± 5.5	0.492	0.050
AFP > 400 ng/mL	15 (3.9)	31 (3.4)	0.763	0.028	14 (3.8)	13 (3.5)	1.000	0.014
PIVKA-II > 40 mAU/mL	158 (41.4)	321 (35.1)	0.039	0.129	151 (40.5)	146 (39.1)	0.765	0.027
Donor variables								
Donor age (years)	31.1 ± 9.0	30.4 ± 8.7	0.165	0.084	31.0 ± 8.9	31.3 ± 9.5	0.662	0.032
Donor sex, male	250 (65.3)	613 (67.1)	0.575	0.038	245 (65.7)	254 (68.1)	0.534	0.051
Total fatty change (%)	3.8 ± 6.1	4.3 ± 6.7	0.240	0.073	3.8 ± 5.9	4.1 ± 5.9	0.480	0.052
Low GRWR (<0.8)	29 (7.6)	109 (11.9)	0.026	0.147	29 (7.8)	33 (8.8)	0.691	0.039
Intraoperative variables								
Anesthesia time (h)	12.9 ± 2.5	13.0 ± 2.3	0.497	0.041	12.9 ± 2.5	12.9 ± 2.2	0.730	0.025
Massive transfusion	105 (27.4)	235 (25.7)	0.570	0.039	99 (26.5)	101 (27.1)	0.934	0.012
Tumor-related variables								
BCLC stage			0.080	0.177			0.992	0.038
Stage 0	164 (42.8)	358 (39.2)			160 (42.9)	161 (43.2)		
Stage A	88 (23.0)	232 (25.4)			88 (23.6)	91 (24.4)		
Stage B	31 (8.1)	108 (11.8)			31 (8.3)	28 (7.5)		
Stage C	54 (14.1)	137 (15.0)			52 (13.9)	53 (14.2)		
Stage D	46 (12.0)	79 (8.6)			42 (11.3)	40 (10.7)		
Within Milan criteria	306 (79.9)	716 (78.3)	0.581	0.038	300 (80.4)	299 (80.2)	1.000	0.007
Within UCSF criteria	325 (84.9)	773 (84.6)	0.964	0.008	317 (85.0)	317 (85.0)	1.000	<0.001
PET (hypermetabolism)	111 (29.0)	261 (28.6)	0.930	0.009	106 (28.4)	108 (29.0)	0.935	0.012
Number of tumor nodules	1.6 ± 2.0	1.6 ± 1.7	0.698	0.023	1.6 ± 2.0	1.4 ± 1.6	0.330	0.071
Maximal tumor size (cm)	1.9 ± 2.0	1.9 ± 1.8	0.899	0.008	1.9 ± 2.0	1.8 ± 1.8	0.465	0.054
Tumor necrosis > 30%	190 (49.6)	466 (51.0)	0.696	0.028	186 (49.9)	186 (49.9)	1.000	<0.001
Vascular or LN invasion	61 (15.9)	148 (16.2)	0.971	0.007	59 (15.8)	58 (15.5)	1.000	0.007

Values are expressed as mean ± standard deviation or number of patients (%) as appropriate. Abbreviations: MELD, Model for End-Stage Liver Disease; AFP, alpha-fetoprotein; PIVKA-II, protein induced by vitamin K absence or antagonist-II; GRWR, graft-to-recipient weight ratio; BCLC, Barcelona Clinic Liver Cancer; UCSF, University of California San Francisco; PET, positron emission tomography; LN, lymph node; SMD, standardized mean difference.

**Table 2:** Incidence and cumulative rates of hepatocellular carcinoma recurrence and all-cause mortality.

	Sevoflurane	Desflurane	*p*
HCC recurrence			
Number of events, *n* (%)	73 (19.6%)	49 (13.1%)	0.023
1-year cumulative recurrence rate (%)	5.3	2.7	<0.001
Incidence rate (per 100 person-years)	13.7	8.7	0.039
Mortality			
Number of events, *n* (%)	58 (15.5%)	58 (15.5%)	1.000
1-year cumulative mortality rate (%)	3.4	4.9	0.307
Incidence rate (per 100 person-years)	3.87	2.93	0.134

Abbreviations: HCC, hepatocellular carcinoma.

**Table 3:** Univariate and multivariate Cox proportional hazards regression analyses for tumor recurrence.

	Univariate Analysis			Multivariate Analysis		
	HR	95% CI	*p*	HR	95% CI	*p*
Anesthetic type (Sevoflurane)	1.77	1.23–2.55	0.002	1.66	1.15–2.39	0.007
Age (years)	1.00	0.98–1.02	0.887	-	-	-
Sex, male	1.54	0.92–2.57	0.100	-	-	-
MELD score	0.99	0.96–1.02	0.520	-	-	-
Donor age (years)	0.99	0.97–1.01	0.258	-	-	-
Donor sex, male	0.94	0.65–1.37	0.746	-	-	-
Massive transfusion	1.00	0.66–1.50	0.982	-	-	-
Beyond Milan criteria	3.44	2.40–4.93	<0.001	-	-	-
AFP > 400 ng/mL	4.02	2.26–7.14	<0.001	-	-	-
PIVKA-II > 40 mAU/mL	2.47	1.72–3.55	<0.001	1.61	1.08–2.40	0.018
PET (hypermetabolism)	3.09	2.17–4.41	<0.001	1.88	1.26–2.79	0.002
Maximal tumor size (cm)	1.32	1.24–1.41	<0.001	1.11	1.02–1.21	0.017
Tumor necrosis > 30%	1.54	1.07–2.22	0.019	1.43	0.98–2.10	0.065
Vascular or LN invasion	5.41	3.77–7.75	<0.001	3.08	2.03–4.67	<0.001
Transplant year	0.95	0.90–1.00	0.059	0.93	0.89–0.98	0.004

Abbreviations: HR, hazard ratio; CI, confidence interval; MELD, Model for End-Stage Liver Disease; AFP, alpha-fetoprotein; PIVKA-II, protein induced by vitamin K absence or antagonist-II; PET, positron emission tomography; LN, lymph node.

**Table 4:** Univariate and multivariate Cox proportional hazards regression analyses for overall survival.

	Univariate Analysis			Multivariate Analysis		
	HR	95% CI	*p*	HR	95% CI	*p*
Anesthetic type (Sevoflurane)	1.35	0.93–1.95	0.114	1.32	0.91–1.91	0.145
Age (years)	1.04	1.02–1.07	0.002	1.05	1.02–1.08	<0.001
Sex, male	1.66	0.96–2.85	0.068	1.47	0.85–2.54	0.171
MELD score	1.02	0.99–1.05	0.214	-	-	-
Donor age (years)	1.01	0.99–1.03	0.295	-	-	-
Donor sex, male	0.92	0.62–1.36	0.670	-	-	-
Massive transfusion	1.19	0.80–1.77	0.399	-	-	-
Beyond Milan criteria	3.77	2.62–5.44	<0.001	2.18	1.43–3.32	<0.001
AFP > 400 ng/mL	2.78	1.50–5.18	0.001	-	-	-
PIVKA-II > 40 mAU/mL	2.16	1.49–3.11	<0.001	-	-	-
PET (hypermetabolism)	2.12	1.47–3.06	<0.001	1.58	1.07–2.35	0.023
Maximal tumor size (cm)	1.22	1.14–1.30	<0.001	-	-	-
Tumor necrosis > 30%	1.04	0.72–1.50	0.828	-	-	-
Vascular or LN invasion	4.39	3.01–6.40	<0.001	2.81	1.84–4.29	<0.001
Transplant year	0.96	0.91–1.02	0.163	-	-	-

Abbreviations: HR, hazard ratio; CI, confidence interval; MELD, Model for End-Stage Liver Disease; AFP, alpha-fetoprotein; PIVKA-II, protein induced by vitamin K absence or antagonist-II; PET, positron emission tomography; LN, lymph node.

## Data Availability

The datasets used and/or analyzed during the current study are available from the corresponding author upon reasonable request.

## Abbreviations

The following abbreviations are used in this manuscript:

AFP	Alpha-fetoprotein
BCLC	Barcelona Clinic Liver Cancer
CI	Confidence interval
CRP	C-reactive protein
GRWR	Graft-to-recipient weight ratio
HCC	Hepatocellular carcinoma
HR	Hazard ratio
LDLT	Living donor liver transplantation
LN	Lymph node
LQMM	Linear quantile mixed model
LT	Liver transplantation
MELD	Model for End-Stage Liver Disease
NK	Natural killer
OS	Overall survival
PET	Positron emission tomography
PIVKA-II	Protein induced by vitamin K absence or antagonist-II
POD	Postoperative day
PSM	Propensity score matching
SMD	Standardized mean difference
TIVA	Total intravenous anesthesia
UCSF	University of California San Francisco
